# Highly Flexible Silicone Coated Neural Array for Intracochlear Electrical Stimulation

**DOI:** 10.1155/2015/109702

**Published:** 2015-07-05

**Authors:** P. Bhatti, J. Van Beek-King, A. Sharpe, J. Crawford, S. Tridandapani, B. McKinnon, D. Blake

**Affiliations:** ^1^School of Electrical and Computer Engineering, Georgia Institute of Technology, Atlanta, GA 30332, USA; ^2^Department of Rehabilitative Medicine, Emory University School of Medicine, Atlanta, GA 30322, USA; ^3^Department of Otolaryngology-Head & Neck Surgery, Georgia Regents University, Augusta, GA 30912, USA; ^4^Department of Neurology, Brain and Behavior Discovery Institute, Georgia Regents University, Augusta, GA 30912, USA; ^5^Department of Radiology and Imaging Sciences, Emory University School of Medicine, Atlanta, GA 30322, USA; ^6^The Shea Ear Clinic, Memphis, TN 38119, USA; ^7^Department of Otolaryngology-Head and Neck Surgery, University of Tennessee Health Sciences Center, Memphis, TN 38163, USA

## Abstract

We present an effective method for tailoring the flexibility of a commercial thin-film polymer electrode array for intracochlear electrical stimulation. Using a pneumatically driven dispensing system, an average 232 ± 64 *μ*m (mean ± SD) thickness layer of silicone adhesive coating was applied to stiffen the underside of polyimide multisite arrays. Additional silicone was applied to the tip to protect neural tissue during insertion and along the array to improve surgical handling. Each array supported 20 platinum sites (180 *μ*m dia., 250 *μ*m pitch), spanning nearly 28 mm in length and 400 *μ*m in width. We report an average intracochlear stimulating current threshold of 170 ± 93 *μ*A to evoke an auditory brainstem response in 7 acutely deafened felines. A total of 10 arrays were each inserted through a round window approach into the cochlea's basal turn of eight felines with one delamination occurring upon insertion (preliminary results of the *in vivo* data presented at the 48th Annual Meeting American Neurotology Society, Orlando, FL, April 2013, and reported in Van Beek-King 2014). Using microcomputed tomography imaging (50 *μ*m resolution), distances ranging from 100 to 565 *μ*m from the cochlea's central modiolus were measured. Our method combines the utility of readily available commercial devices with a straightforward postprocessing step on the order of 24 hours.

## 1. Introduction

Since their introduction in the mid 1970s [1] planar thin-film arrays (TFAs) have become an invaluable scientific tool for systems neurophysiology. Through the utilization of integrated circuit fabrication methods, TFAs have been developed on a variety of substrates including sapphire, metal, glass, silicon, and polymers [1–5]. By layering and patterning dielectrics and conductors upon such substrates, multisite stimulating and recording electrode arrays have been realized with submicron precision. Serving as an essential interface between the nervous system and microelectronics, TFAs have enabled a greater understanding of electrical and chemical signaling in the brain [6–9], as well as a therapeutic option for overcoming sensory loss in the auditory [10–13] and visual [14, 15] systems. The ongoing validation, as well as the need, of multisite TFAs has ultimately led to the commercial availability of lithographically defined, batch-processed arrays (Neural Nexus Technologies, Ann Arbor, MI). Primarily based on silicon or polyimide substrates, these arrays can be custom-designed or selected from a design library, for neurophysiological studies.

While the availability of commercial TFAs alleviates the time-consuming burden of fabrication, many* in vivo* applications require some amount of postprocessing to enable a TFA to physically approach anatomic structures. One such example is the cochlea: the average human cochlea rotates through two and one half turns from base to apex and is comprised of three chambers, or scala. The efficacy of a silicon-based TFA for intracochlear stimulation has been reported in animal models [11, 16]. However, such arrays have proven difficult to insert and accurately place in the lower chamber (scala tympani) during acute* in vivo* studies. Given the stiffness of the silicon-based TFAs, a* flexible* TFA alternative is essential. Pursuing such an alternative we investigated flexible, biocompatible polyimide based TFAs for intracochlear stimulation. Our first postprocessing approach involved mechanically adhering a TFA to an insertion platform (IP) that is similar to commercial intracochlear arrays [17, 18]. Results were mixed in human cadaver insertion studies with TFA-IP devices (*n* = 10). Two delaminations, where the array separated from the IP, occurred. Moreover, microcomputed tomography imaging (50 *μ*m resolution) revealed undesirable placement of two devices [19]. One TFA-IP was placed in the semicircular canal and one in the vestibule, indicating that significant improvement of the TFA-IP integration strategy was essential.

In this paper, we extend the applicability of TFAs for intracochlear stimulation through a simple and effective method. The robustness of a commercial thin-film polyimide intracochlear electrode array can be improved by layering silicon adhesive to the underside of the TFA. Furthermore, additional silicone handling points along the TFA may be provided for the surgeon since insertion requires force to advance the array along the cochlea [20–24]. We also discuss the method for constructing such arrays and report results of intracochlear stimulation using these arrays in acute feline studies.

## 2. Methods

### 2.1. Electrode Array Modification

**Figure 1:**
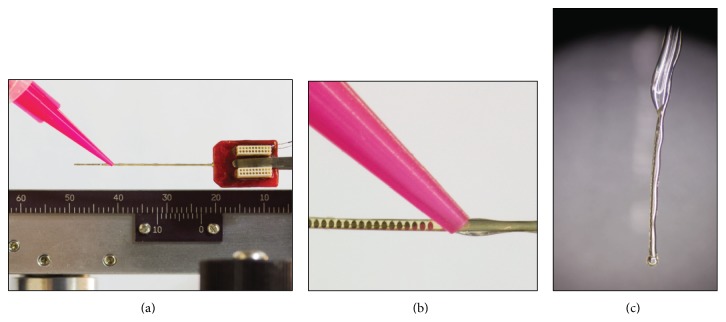
TFA and coating procedure. (a) TFA measured 27.8 mm × 0.4 mm × 20 *μ*m (*L* × *W* × *H*) with 20 functional platinum sites (180 *μ*m diameter, 250 *μ*m pitch). Two 16-channel Omnetics connectors bonded to the backend of each array enable signal transfer. (b) Topside application of silicone adhesive. (c) Final silicone coated TFA (cTFA). Note silicone ball at distal tip to reduce the potential for insertion trauma.

Polyimide thin-film arrays were custom-designed for insertion into the basal turn of the cochlea and microfabricated by a commercial foundry (NeuroNexus Technologies). Composed of gold conducting traces sandwiched between layers of polyimide (Figure 1), each TFA measured 27.8 mm × 0.4 mm × 20 *μ*m (*L* × *W* × *H*) and supported 21 platinum sites (180 *μ*m dia., 250 *μ*m pitch). For signal transfer, the array manufacturer provided two 16-channel connectors (Omnetics Connector Corp., Minneapolis, MN) bonded to the backend of each array.

Layers of MED-2000 Silicone RTV Adhesive (NuSil Silicone Technology LLC, Carpinteria, CA) were applied to the top and bottom surfaces of each flexible TFA (Young's modulus, 3 GPa) to provide the needed rigidity for insertion (Figure 1(a)). Each array was affixed to a micromanipulator and suspended vertically. A digitally controlled pneumatic dispenser system (Madell Technology Corp., Ontario, CA), with a 25-gauge plastic tip at a pressure of 25 PSI, was then used to dispense a layer of silicone adhesive along the entire underside length of the TFA. This was done carefully, in six-second intervals, ensuring that the adhesive was only applied to the bottom of the TFA. To compensate for the force generated by the needle applicator, a solid supporting edge was placed on the opposing side of the suspended array. The adhesive set quickly, mitigating any effects of gravity drawing the glue down. The TFA remained suspended in the micromanipulator for one hour to dry further. The TFA was then placed on a clean glass slide with the existing adhesive layer facing down to position the TFA for application of the second layer on the topside of the TFA (Figure 1(b)). Using a bench-top low-power microscope, additional adhesive was applied from the connector base down the length of the array within 1 mm of the active sites to further support the TFA. For this application, the dispenser tip was replaced with a 22-gauge tip and the pressure adjusted to 20 PSI. Applying a wider strip of adhesive enabled the adhesive to flow around the width of the array and bond to the rear layer, thus fully securing the array in silicone. Finally, a small amount of adhesive was added to the array tip that covered site 1 (most distal site). The goal was to help distribute insertion forces and reduce insertion trauma. The completed coated TFA (cTFA) array was left to cure for 24 hours (Figure 1(c)).

Sites were inspected visually and validated functionally by measuring impedances in phosphorous-buffered saline solution (PBS). A custom connector box with cables was made to access the two 16-channel connectors on the back end of the cTFA. To test site impedance, a ±100 nA 1 kHz sinusoidal signal was applied independently to each site with a PlexStim 2.0 (Plexon Inc., Dallas, TX) 16-channel stimulator. The peak voltage for each site was measured using a Hameg HM507 oscilloscope and used to compute site impedance.

### 2.2. Surgical Procedure

The study was a prospective cochlear array insertion analysis with electrically evoked auditory brainstem response testing in a feline model, using previously published feline implantation techniques [25–27]. Approval was obtained from the Georgia Regents University and the Georgia Institute of Technology Institutional Animal Care and Use Committees (2011-0362, A12086). One resident veterinarian and one veterinarian technician were present for surgical preparation and throughout the experiment to monitor the subject. The surgical team included a board-certified otolaryngologist with 12 years of experience and a third-year otolaryngology resident. Eight healthy, adult wild-type felines weighing ≥ 3 kg (4 females and 4 males) were used as the subjects for all* in vivo* implantation and electrical stimulation tests. All proper quarantine protocols were strictly followed. Subjects were randomly assigned to undergo implantation of the right or left ear with a cTFA. The contralateral unimplanted ear of each feline served as the control for that feline. Intravenous access was obtained in a vein from one of the front legs. The subjects were anesthetized with intravenous ketamine : medetomidine (5 : 0.05 mg/kg) for induction, followed by endotracheal intubation for ventilation and isoflurane (1–3%). Later experiments used an intravenous acepromazine : butorphanol (0.1 : 0.3 mg/kg) induction, followed by propofol (8–10 mg/kg), followed by endotracheal intubation for ventilation, and isoflurane (1–3%). For the duration of the surgery, heart rate, respiratory rate, oxygen saturation, and end-tidal carbon dioxide were continually monitored and body temperature was maintained at 38°C using a controlled heating pad. Subjects received continuous infusion of intravenous Ringer's Lactate solution with 2.5 percent dextrose at a rate of 10 mL : kg : hr.

Preoperative auditory brainstem response (ABR) was obtained to ensure bilateral hearing after induction of general anesthesia, but prior to any surgical intervention. To verify the baseline, recordings were obtained using silver wires as electrodes inserted into the vertex of the scalp using a 22-gauge needle. Signals were measured differentially between ipsilateral bulla and vertex with the contralateral bulla as the ground for the control ear of each subject. The differential signal was AC coupled, amplified by 10 k, and bandpass-filtered over a 200 Hz to 10 kHz frequency range. The resulting analog signal was then converted to a digital signal using a 16-bit analog-to-digital converter at a 10 kHz sampling rate. A total of 1000 repetitions were averaged. A National Instruments (National Instruments Inc., Austin, TX) card with limits ±5 V served as the instrument control interface. Condensation acoustic clicks, 0.01 msec, were applied with a distance of 12 inches from the subject at a sound level of less than or equal to 80 dB SPL in one ear with the contralateral ear plugged. Waveforms were recorded for analysis using custom software written in LabView (National Instruments, Inc.). Click-evoked ABRs down to 60 dB SPL were confirmed in every case. A foam earplug was placed in the contralateral ear to avoid any interference of eliciting a response from the normal hearing ear during electrical stimulation.

The surgical approach to the cochlea was similar to that in humans, with feline specifications [25–27]. One of two surgeons performed each insertion. After palpating surgical landmarks including the temporal line and the posterior aspect of the external auditory canal, a C-shaped incision was made behind the randomly chosen ear. The outer ear canal was exposed and dissection continued to the osseous skull. An approximately 5 × 5 mm bullectomy was drilled to gain access to the cochlea under magnification. Perilymph was removed via wicking through the cochleostomy and replaced with 10 percent neomycin sulfate solution to induce acute hearing loss. After two minutes, fluid was once again wicked from the basal turn of the cochlea and replaced with 10 percent neomycin to acutely deafen the feline. This procedure was repeated until the lack of ABRs at 60 dB SPL was confirmed after neomycin treatments.

A cTFA was manually inserted into the scala tympani through the round window. Insertion was performed with microscopy and the array was advanced until some resistance was perceived by the surgeon. The cTFA was secured in place with a hemostat to avoid movement during electrical testing.

### 2.3. Electrical Testing Protocol

After insertion of the array, at least five site impedances along the length of the array were sampled. This was to detect if any transverse breaks along the cTFA occurred during insertion. Similar to the* in vitro* impedance testing mentioned above, a ±100 nA 1 kHz sinusoidal signal was applied with the PlexStim 2.0 Stimulator between each site and a 22-gauge needle ground electrode inserted into the local subcutaneous tissue behind the ipsilateral ear.

**Figure 2:**
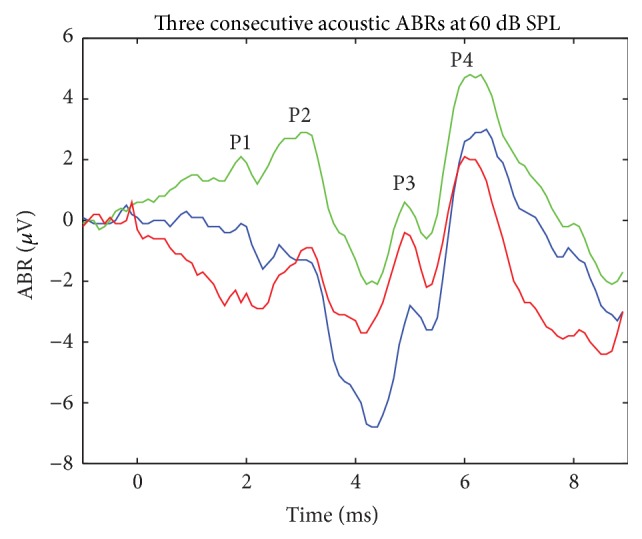
Click-evoked auditory brainstem response at 60 dB SPL. The responses were averaged over 1000 repetitions. A 1 msec delay occurs due to placement of the speaker 12 inches from the ear. Three consecutive responses are plotted (order: blue, green, and red).

Monopolar electrical stimulation was applied between an intracochlear cTFA electrode and the ground return electrode. Sites spanning the entire array length were tested in a random order. Triggered by a TTL output pulse from the ABR recording instrument control, all stimuli were charge-balanced biphasic pulses, negative first, and balanced with a positive phase, (200 *μ*sec per phase). An interphase gap of 10 *μ*sec was applied during which the electrodes were grounded to prevent charge build-up. Applied stimulation current ranged from 100 to 500 *μ*A in magnitude. Stimulus artifact was subtracted out by averaging with stimulation at an inverted phase. Stimulation occurred at 5–10 Hz and up to 1000 trials. The resulting electrically evoked auditory brainstem response (eABR) signal was processed following the same procedure as the ABR signal. For each site, threshold was determined as the level of applied current just below an appreciable eABR. Assessed visually by the attending electrophysiologist, this occurred when the eABR response failed to evoke a triple peaked auditory brainstem response analogous to the acoustically evoked auditory brainstem response. EABR stimuli were repeated until a definitive assessment was made by the electrophysiologist. The specific details of the testing protocol are as follows. For the first site tested on each array, a stimulus midway between 100 *μ*A and 500 *μ*A was applied (300 *μ*A). If an eABR was observed at the starting point of 300 *μ*A, as confirmed by 2-3 trials, a stimulus level midway between 300 *μ*A and 100 *μ*A was applied (200 *μ*A). Upon eABR confirmation, a lower stimulus level midway between 200 *μ*A and 100 *μ*A was applied to the site (150 *μ*A). For the lowest bound, sub-100 *μ*A stimuli were not applied since a threshold of 100 *μ*A or less was considered to represent the normal-low range. Once an eABR was lost, as confirmed by the loss of peaks P3 and P4 (Figure 2), the current was increased until the best peak 4-5 msec into the plot was observed over multiple runs. This current level was assigned as the threshold value for the site under test. In the opposite direction, a similar procedure was followed with 500 *μ*A as the upper bound. For subsequent sites on a given array, testing began with the threshold value of the previous site as a starting point. The stimulus level was increased and decreased in a similar fashion described above by bisecting the interval between the immediate value and the upper or lower bound. Given the variability in ABRs in general, the precision in threshold values is estimated as 50 *μ*A. When eABR stimuli failed to evoke a triple peaked (Waves P2–P4; see Figure 2) auditory brainstem response over the entire applied current range for at least two trials, the eABR was recorded as indeterminate for the site under testing.

To validate electrode site functionality and examine impedance changes due to stimulation, poststimulation impedance values were determined similar to prestimulation impedance testing.

### 2.4. Computed Tomography Imaging

Two cTFAs were independently imaged in a microcomputed tomography (CT) system, and one cTFA was imaged in a harvested feline cochlea after the electrical testing was completed. Imaging studies were conducted at the Emory School of Medicine's Center for Systems Imaging, Atlanta, GA, using a Siemens Inveon MicroPET:CT Preclinical Scanner (Siemens Medical Solutions USA, Inc.; Hoffman Estates, IL). The MicroCT images were made with a pixel size of 20 × 20 *μ*m, slice thickness 21.5 *μ*m, and resolution of 46.499 pixels per mm. Total slice size was 1152 × 1152 pixels (24.77 × 24.77 mm). The volume data was reoriented so that the distal end of the array was in the image plane. Measurements were made using OsiriX MD (Pixmeo SARL; Geneva, Switzerland) digital imaging software by a board-certified radiologist.

## 3. Results

### 3.1. Electrode Array Impedance Measurements

A total of 19 TFAs were coated. For each TFA, all site impedance values at 1 kHz were provided by the manufacturer to demonstrate site viability (one site each on four TFAs sites was nonfunctional as indicated by the manufacturer). The average of all site impedances was 90.4 ± 36.3 kΩ (mean ± SD). Postsilicone adhesive application site impedances (for the first 10 cTFAs tested, only the first 16 sites and the 21st site were electrically accessible due to routing error in the custom connector box. For the next 9 cTFAs all sites were accessible) measured in PBS demonstrated an average value of 119.9 ± 92.4 kΩ. The tip site (site 0) was not considered in the calculation given that it was coated with silicone to reduce insertion trauma. For each cTFA, sites that presented an* in vitro* impedance greater than 500 kΩ were considered nonfunctional resulting in 7 percent of sites deemed as nonfunctional (24 out of 346 sites).

### 3.2. Intracochlear Electrical Stimulation

All eight felines had baseline hearing documented by ABR. Figure 2 illustrates three consecutive ABRs at 60 dB SPL labeled with the appropriate positive response peaks [28]. A 1 msec delay occurs due to the placement of the speaker at 12 inches from the ear. The noise is small with a substantial artifact on some runs, and these occur in different positions. However, consistency is observed in multiple plots. Thus the data appears to be statistically well behaved.

Feline demographics are listed in Table 1. Unilateral deafening was achieved following four to eight applications of 10 percent neomycin. Following this, normal hearing was documented in unimplanted ears. All felines had subjectively easy, full insertion of the cTFA, with full insertion established at point of first resistance.

Ten of the 19 cTFAs were used* in vivo *for electrical stimulation. No sites were lost during the stimulation process. One delamination, where the silicone coating separated from the TFA, was observed upon cTFA removal. In this particular animal (feline 3) facial nerve activation was observed and is discussed below.

The average* in vivo* site impedances were 184.5 ± 147.9 kΩ before stimulation and 67.1 ± 43.3 kΩ after stimulation. To further examine site impedance trends the cTFA was divided into three physical segments with reference to the insertion point: proximal, central, and distal (Table 2). Note that each cTFA was inserted into the basal turn only. Thus the segments represent more basal, central, and more apical sites, in the basal turn itself. For each segment, poststimulation site impedances were measured for one to three sites and averaged per cTFA as well as across all inserted cTFAs per segment (Table 2).

**Figure 3:**
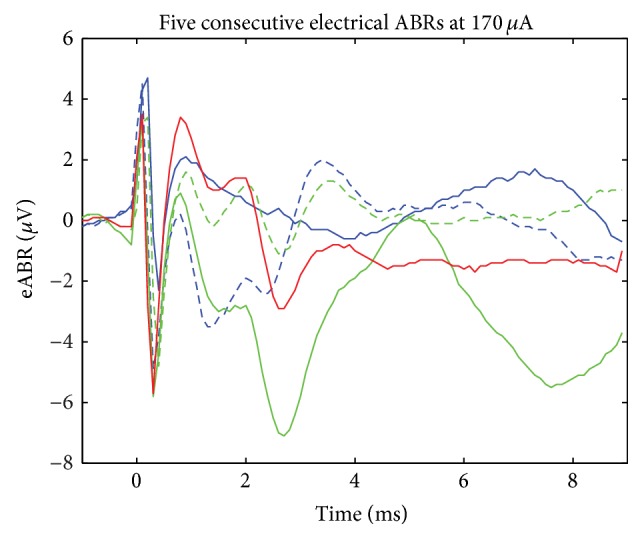
Electrically evoked auditory brainstem responses. The first 0–0.5 msec is artifact. The following 0.8–5 msec is electrical ABR. Based on 500 repetitions, the threshold as indicated by the eABR is 170 *μ*A of monopolar current. Five consecutive responses are plotted (order: blue, blue dash, green, green dash, and red) illustrating the range of observations.

**Figure 4:**
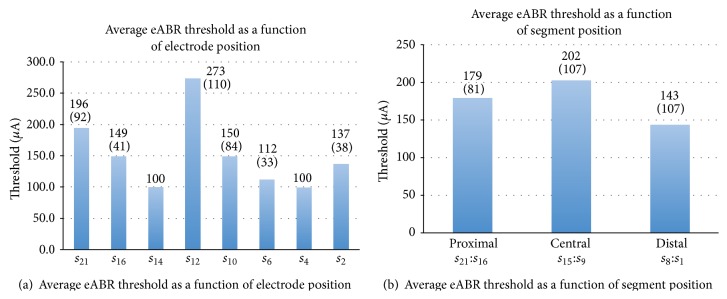
(a) Average of eABR as a function of site position for all arrays tested (*n* = 7). (b) Average of eABR as a function of segment for all arrays tested (*n* = 7). Standard deviation in parentheses.

**Figure 5:**
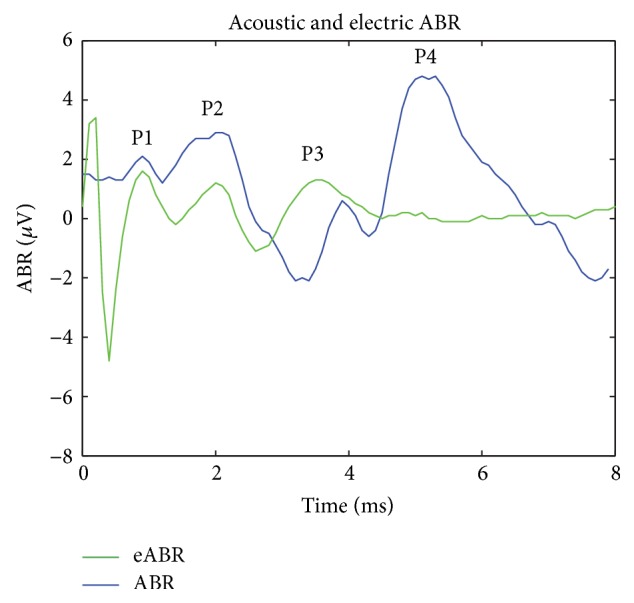
Composite of acoustically and electrically evoked auditory brainstem responses at 60 dB SPL and 170 *μ*A, respectively. Acoustic ABR shifted by 1 msec to account for delay from speaker 12 inches from ear.

Five consecutive eABRs are shown in Figure 3. Using the eABR as a guide, the average intracochlear stimulating current threshold was 170 ± 93 *μ*A. The per-array thresholds and per-segment thresholds, which are summarized in Figures 4(a)-4(b), further illustrate the per-segment thresholds as well as per-site thresholds across all arrays tested. For comparison, Figure 5 illustrates a composite of acoustic (60 dB SPL) and electrically (170 *μ*A) evoked auditory brainstem responses. The acoustic ABR is shifted by 1 msec due to the placement of the speaker 12 inches from the ear.

In one animal, facial nerve twitching was observed with 400 *μ*A biphasic stimulation at site six. The stimulator was disconnected immediately from the animal. To investigate if the nerve twitching was segment specific, sites in each segment were stimulated. Two outcomes were observed, twitching at approximately 200 *μ*A, or no eABR at approximately 100 *μ*A. The cTFA was explanted and delamination was observed. A second array was then inserted into the same animal and tested but eABRs were not observed.

### 3.3. Computed Tomography Imaging

Two cTFAs were imaged independently to measure silicone coating thickness. Topside coating thickness was measured at 1–1.5 mm intervals averaging 357 ± 81 *μ*m. Bottom-side coating, under the sites, was measured at 500 *μ*m intervals and averaged 232 ± 64 *μ*m in thickness. One of the cTFAs was imaged after insertion revealing no structural changes.

**Figure 6:**
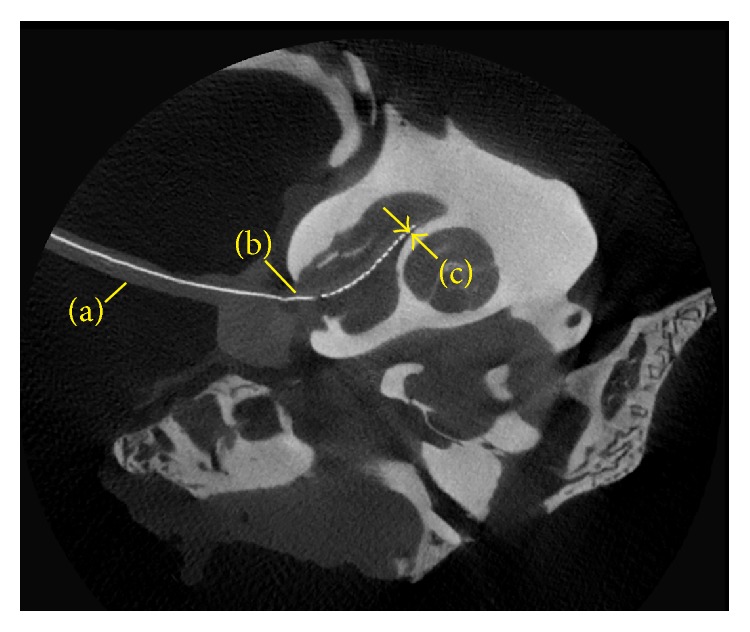
cTFA insertion into the basal turn. (a) Electrode array, (b) round window, (c) at approximately site 8; a distance of 100 microns from the modiolus was measured.

The cTFA in feline 4 was imaged to observe placement (Figure 6), illustrating insertion through the round window and into the base. A maximum distance of 565 *μ*m from the modiolus and a minimum distance of 100 *μ*m at approximately site 8 were measured. Since the angle of insertion into the cochlea cannot be accurately measured, the true depth of insertion cannot be calculated accurately. There was trauma to the osseous spiral lamina with the tip of the electrode in the scala vestibuli.

## 4. Discussion and Conclusion

This paper demonstrates a simple, cost-effective method to adjust the flexibility of fabricated polymeric thin-film arrays. The addition of a medical grade silicone coating enabled (1) insertion of a research array via the round window approach into the basal turn of the cochlea and (2) the associated electrical activation of the central auditory system. From a mechanical standpoint the cTFAs were not compromised during insertion or stimulation. This was validated by prestimulation and postsite impedance; that is, no open circuits were found. Considering the site impedance values, the observed* in vivo* impedances were appreciably high when compared with contemporary electrode arrays (typically 10–20 kΩ). This may be in part attributed to dramatically reduced site area. The high-density arrays present a site area of 0.025 mm^2^, nearly one order of magnitude smaller than contemporary arrays. With respect to the silicone coating, only one delamination occurred. In situ imaging of one TFA gave no indication of delamination or localized separation of the coating from the TFA.

During the process of electrical stimulation, poststimulation site impedance varied across the arrays with the smallest average and standard deviation occurring in the central array segment. When compared with commercial arrays, the cTFAs were not tapered to follow the widening of the base or the narrowing toward the apex. Possibly, the central sites that are the closest to the modiolus (Figure 6) demonstrated the largest impedance values. Additionally, similar to human implantations, it is likely that there was some variability in insertion depth.

Electrically evoked auditory brainstem response waveforms were consistent with those reported for the feline model previously [29, 30]. Threshold data exhibited considerable variability with no obvious correlation to a segment or site. Nonetheless, in some of the animals tested, a pronounced distal versus proximal threshold effect was observed.

A potential challenge is the insertion. Intracochlear insertion trauma is an important concern as the minimization of trauma is essential for hearing preservation techniques. In previous work using cadaveric human temporal bones, the percentage trauma was 26 percent overall for all TFA electrode insertions (30 percent for the cochleostomy and 22 percent for the round window approaches), though interestingly, intracochlear trauma due to embalming and cold storage was noted in 29 percent of controls not implanted [17]. Evaluation of trauma using the Cochlear (New South Wales, Australia) devices showed that intracochlear trauma was found in 16 percent of cochlea implanted with the banded electrode arrays and 13 percent of cochlea implanted with the contour [20], on average. The Advanced Bionics (Valencia, CA) Spiral, a precurved, perimodiolar design, appeared to cause intracochlear trauma in 9.5 percent of cochlea undergoing short insertion (<400°) and intracochlear trauma in 31 percent undergoing insertion the entire electrode length. The incidence of intracochlear trauma with the HiFocus II was 8.3 percent during the short insertion and 44.5 percent trauma during full electrode insertion [21].

While possible scala vestibule placement was seen in the single feline cochlea imaged, based on past experience with this design, intracochlear insertion trauma is expected to be less than that seen with other electrode designs. Furthermore, while the placement is suboptimal, there were no detectable changes to the silicone coating or TFA. This suggests that the silicone coating can withstand surgical handling as the cTFA is advanced into the cochlea. Undoubtedly, contemporary electrode arrays as part of commercial cochlear implant systems demonstrate reliable insertion and placement. However, they are constructed by hand and consist of wire bundles encased in silicone and are therefore difficult to modify.

Looking toward the future, to explore advanced stimulation strategies with more electrode sites [31], these arrays cannot simply be scaled up. The resulting array size precludes insertion past the second turn of the scala tympani, which narrows to 200 *μ*m in humans [32]. In contrast, the microfabrication process for TFAs enables electrode site densities to expand two to three times more than commercial devices while remaining within the minimum scalar dimension. Furthermore, when considering the development of combined acoustical and electrical stimulation for patients with residual hearing in the low frequencies, hearing preservation is paramount. Studies have indicated that thinner arrays are associated with lower postimplantation hearing thresholds in animal models [33], and thinner tips (250–350 *μ*m) are associated with lower insertion forces during implantation [34].

The method of coating thin-films with silicone reported in this work could be refined to a repeatable manufacturing method. For example, layers of silicone could be molded onto the TFAs in consistent thicknesses and exact locations. Furthermore, modulating the substrate flexibility could possibly enable the application of existing neural recording and stimulating arrays to more reliably probe spaces and expand our understanding of signaling and modulation in the cochlea and beyond.

## Figures and Tables

**Table 1:** Summary of feline demographics and average eABR threshold.

Feline	1	2	3	4	5	6	7	8
Sex	Female	Female	Female	Male	Male	Male	Male	Female
Weight (kg)	3.80	4.64	4.29	6.67	5.31	4.47	4.68	3.42
Side	Left	Left	Left	Right	Right	Right	Right	Right
eABR threshold mean ± SD (*μ*A)	273 ± 133	206 ± 59	N:A *Facial nerve stimulation *	119 ± 21	145 ± 37	100 ± 25	141 ± 46	228 ± 93

**Table:** (a)


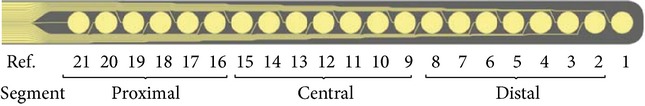												

	Proximal segment			Central segment				Distal segment				

Feline 1												
Site number	21	16		12		10		6		2		
Impedance (kΩ)	44.6	NT		40.0		36.4		35.6		36.6		
Mean (kΩ)	43.6				38.2				36.1			38.4 ± 3.4
Feline 2												
Site number	21	16		12		10		6		2		
Impedance (kΩ)	44.0	38.0		42.0		NT		44.0		36.0		
Mean (kΩ)	41.0				42.0				40.0			40.8 ± 3.6
Feline 3												
Site number	21	16		12		10		6		2		
Impedance (kΩ)	52.0	196.0		NT		34.0		128.0		56.0		
Mean (kΩ)	124.0				34.0				92.0			93.2 ± 67.8
Feline 4												
Site number	21	16		12		10		6		2		
Impedance (kΩ)	76.0	48.0		NT		50.0		52.0		48.0		
Mean (kΩ)	62.0				50.0				50.0			54.8 ± 11.9
Feline 5												
Site number	21	16		12		10		6		2		
Impedance (kΩ)	64.0	68.0		NT		156.0		156.0		180.0		
Mean (kΩ)	66.0				156.0				168.0			124.8 ± 54.6
Feline 6												
Site number	21	16		12	10	8		6	4	2		
Impedance (kΩ)	40.0	44.0		48.0	48.0	48.0		52.0	52.0	48.0		
Mean (kΩ)	52.0				48.0				50.7			47.5 ± 3.9
Feline 7												
Site number	20	14		12		10		6		2		
Impedance (kΩ)	65.0	145.0		NT		90.0		50.0		60.0		
Mean (kΩ)	105.0				90.0				55.0			82.0 ± 38.2
Per segment mean (kΩ)	71.0 ± 46.9			59.2 ± 37.4				68.9 ± 45.9				

**(b):** 


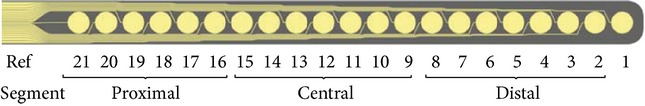												

	Proximal segment			Central segment				Distal segment				

Feline 1												
Site number	21	16		12		10		6		2		
Threshold (*μ*A)	^*^ind	NT		450		300		170		170		
Mean (*μ*A)	^*^ind				375				170			273 ± 133
Feline 2												
Site number	21	16		12		10		6		2		
Threshold (*μ*A)	300	220		190		NT		150		170		
Mean (*μ*A)	260				190				160			206 ± 59
Feline 3												
Site number	21	16		12		10		6		2		
Threshold (*μ*A)	125	150		NT		100		100	120			
Mean (*μ*A)	137.5				100					110		119 ± 21
Feline 4												
Site number	21	16		12		10		6		2		
Threshold (*μ*A)	100	150		NT		100		150		1125		
Mean (*μ*A)	125				100				137.5			145 ± 37
Feline 5												
Site number	21	16		12		10		6		2		
Threshold (*μ*A)	125	125		NT		100		75		75		
Mean (*μ*A)	125				100				75			100 ± 25
Feline 6												
Site number	21	16		12	10	8		6	4	2		
Threshold (*μ*A)	^*^ind	^*^ind		180	200	^*^ind		125	100	100		
Mean (*μ*A)	^*^ind				190				108.3			141 ± 46
Feline 7												
Site number	20	14		12		10		6		2		
Threshold (*μ*A)	320	NT		100		200		320		200		
Mean (*μ*A)	320				150				260			228 ± 93
Per segment mean (*μ*A)	179 ± 81			202 ± 107				143 ± 61				

Based on image above, NT = not tested and ^*^ind = indeterminate response to eABR stimuli.

## References

[1] Wise K. D., Angell J. B. (1975). A low capacitance multielectrode probe for use in extracellular neurophysiology. *IEEE Transactions on Biomedical Engineering*.

[2] Sonn M., Feist W. M. (1974). A prototype flexible microelectrode array for implant prosthesis applications. *Medical and Biological Engineering*.

[3] White R. L., Schinder R. A., Merzenich M. M. (1985). Stanford cochlear prosthesis system: ten years of evolution. *Cochlear Implants*.

[4] Clark G. M., Hallworth R. J. (1976). A multiple-electrode array for a cochlear implant. *The Journal of Laryngology & Otology*.

[5] Shamma-Donoghue S. A., May G. A., Cotter N. E., White R. L., Simmons F. B. (1982). Thin film multielectrode arrays for a cochlear prosthesis. *IEEE Transactions on Electron Devices*.

[6] Csicsvari J., Jamieson B., Wise K. D., Buzsáki G. (2003). Mechanisms of gamma oscillations in the hippocampus of the behaving rat. *Neuron*.

[7] Snyder R. L., Bierer J. A., Middlebrooks J. C. (2004). Topographic spread of inferior colliculus activation in response to acoustic and intracochlear electric stimulation. *Journal of the Association for Research in Otolaryngology*.

[8] Johnson M. D., Franklin R. K., Gibson M. D., Brown R. B., Kipke D. R. (2008). Implantable microelectrode arrays for simultaneous electrophysiological and neurochemical recordings. *Journal of Neuroscience Methods*.

[9] Vetter R. J., Williams J. C., Hetke J. F., Nunamaker E. A., Kipke D. R. (2004). Chronic neural recording using silicon-substrate microelectrode arrays implanted in cerebral cortex. *IEEE Transactions on Biomedical Engineering*.

[10] Bell T. E., Wise K. D., Anderson D. J. (1998). A flexible micromachined electrode array for a cochlear prosthesis. *Sensors and Actuators, A: Physical*.

[11] Bhatti P. T., Wise K. D. (2006). A 32-site 4-channel high-density electrode array for a cochlear prosthesis. *IEEE Journal of Solid-State Circuits*.

[12] Wang J., Wise K. D. (2009). A thin-film array with integrated position sensing. *IEEE Journal of Micro Electro Mechical Systems*.

[13] Lim H. H., Anderson D. J. (2007). Spatially distinct functional output regions within the central nucleus of the inferior colliculus: implications for an auditory midbrain implant. *Journal of Neuroscience*.

[14] Kelly S. K., Shire D. B., Chen J. (2011). A hermetic wireless subretinal neurostimulator for vision prostheses. *IEEE Transactions on Biomedical Engineering*.

[15] Humayun M. S., Dorn J. D., Da Cruz L. (2012). Interim results from the international trial of second sight's visual prosthesis. *Ophthalmology*.

[16] Wise K. D., Bhatti P. T., Wang J., Friedrich C. R. (2008). High-density cochlear implants with position sensing and control. *Hearing Research*.

[17] Iverson K. C., Bhatti P. T., Falcone J., Figueroa R., McKinnon B. J. (2011). Cochlear implantation using thin-film array electrodes. *Otolaryngology—Head and Neck Surgery*.

[18] Sharpe A., van Beek-King J., Crane A., McKinnon B., Bhatti P. Integration of a polymeric thin-film array with an insertion platform for a high-density cochlear electrode array.

[19] Bhatti P. T., Van Beek-King J., Tridandapani S., Olsen K., Iverson K., McKinnon B. An Integrated thin-film high-density intracochlearelectrode array and insertion platform: in-vitro validation and temporal bone insertion.

[20] Wardrop P., Whinney D., Rebscher S. J., Roland J. T., Luxford W., Leake P. A. (2005). A temporal bone study of insertion trauma and intracochlear position of cochlear implant electrodes. I: comparison of Nucleus banded and Nucleus Contour electrodes. *Hearing Research*.

[21] Wardrop P., Whinney D., Rebscher S. J., Roland J. T., Luxford W., Leake P. A. (2005). A temporal bone study of insertion trauma and intracochlear position of cochlear implant electrodes. II: comparison of Spiral Clarion and HiFocus II electrodes. *Hearing Research*.

[22] Biedron S., Prescher A., Ilgner J., Westhofen M. (2010). The internal dimensions of the cochlear scalae with special reference to cochlear electrode insertion trauma. *Otology & Neurotology*.

[23] Rebscher S. J., Hetherington A. M., Snyder R. L., Leake P. A., Bonham B. H. (2007). Design and fabrication of multichannel cochlear implants for animal research. *Journal of Neuroscience Methods*.

[24] Rebscher S. J., Hetherington A., Bonham B., Wardrop P., Whinney D., Leake P. A. (2008). Considerations for design of future cochlear implant electrode arrays: electrode array stiffness, size, and depth of insertion. *Journal of Rehabilitation Research and Development*.

[25] Rajguru S. M., Matic A. I., Robinson A. M. (2010). Optical cochlear implants: evaluation of surgical approach and laser parameters in cats. *Hearing Research*.

[26] Kretzmer E. A., Meltzer N. E., Haenggeli C.-A., Ryugo D. K. (2004). An animal model for cochlear implants. *Archives of Otolaryngology—Head & Neck Surgery*.

[27] Kirby A. E., Middlebrooks J. C. (2010). Auditory temporal acuity probed with cochlear implant stimulation and cortical recording. *Journal of Neurophysiology*.

[28] Ungan P., Yagcioglu S. (2002). Origin of the binaural interaction component in wave P4 of the short-latency auditory evoked potentials in the cat: evaluation of serial depth recordings from the brainstem. *Hearing Research*.

[29] Beitel R. E., Snyder R. L., Schreiner C. E., Raggio M. W., Leake P. A. (2000). Electrical cochlear stimulation in the deaf cat: comparisons between psychophysical and central auditory neuronal thresholds. *Journal of Neurophysiology*.

[30] Smith Z. M., Delgutte B. (2007). Using evoked potentials to match interaural electrode pairs with bilateral cochlear implants. *Journal of the Association for Research in Otolaryngology*.

[31] Goldwyn J. H., Bierer S. M., Bierer J. A. (2010). Modeling the electrode-neuron interface of cochlear implants: effects of neural survival, electrode placement, and the partial tripolar configuration. *Hearing Research*.

[32] Erixon E., Högstorp H., Wadin K., Rask-Andersen H. (2009). Variational anatomy of the human cochlea: implications for cochlear implantation. *Otology and Neurotology*.

[33] Nguyen Y., Miroir M., Kazmitcheff G. (2012). Cochlear implant insertion forces in microdissected human cochlea to evaluate a prototype array. *Audiology and Neurotology*.

[34] Nguyen Y., Mosnier I., Borel S. (2013). Evolution of electrode array diameter for hearing preservation in cochlear implantation. *Acta Oto-Laryngologica*.

